# Effects of human Toll-like receptor 1 polymorphisms on ageing

**DOI:** 10.1186/1742-4933-10-4

**Published:** 2013-02-07

**Authors:** Peter Uciechowski, Eva Maria Oellig, Erminia Mariani, Marco Malavolta, Eugenio Mocchegiani, Lothar Rink

**Affiliations:** 1 Institute of Immunology, Medical Faculty, RWTH Aachen University, Pauwelsstr. 30, D-52074, Aachen, Germany; 2 Laboratory of Immunorheumatology and Tissue Regeneration/RAMSES, Codivilla-Putti Research Institute, IOR, Bologna and DIMEC, Bologna University, Bologna, Italy; 3 Immunology Centre, Nutrition, Immunity, and Aging Section, Research Department, INRCA, Ancona, Italy

**Keywords:** Ageing, TLR1, SNP, Nonagenarians, Cytokines, CCL2, IL-1Ra

## Abstract

**Background:**

Advanced age results in crucial alterations of the innate and adaptive immune system leading to functional defects resulting in infection and chronic diseases. Toll-like receptors (TLR) recognize pathogenic structures and are important in the immune response to infections and vaccination. However, the role of TLR single nucleotide polymorphisms (SNP) is poorly understood in the setting of human ageing. This study investigated the impact of the TLR1 SNPs A743G and T1805G on ageing in different age groups from two European populations.

**Results:**

The TLR1 genotypes 743AA/1805GG (TLR1neg) are associated with a TLR1 negative phenotype, impaired function and susceptibility to tuberculosis. Carriers of heterozygous 743AG/1805TG and homozygous 743GG/1805TT genotypes (TLR1pos) have a TLR1 positive phenotype. By comparing healthy young and old German donors, the old group showed a tendency to carry more TLR1neg and less homozygous TLR1pos genotypes. Anti-inflammatory Interleukin (IL)-1 receptor antagonist (Ra) was significantly elevated in supernatants of mononuclear cells from old German subjects with a TLR1pos genotype in contrast to those with the 743AA genotype. Healthy old individuals and nonagenarians from Italy displayed significantly higher frequencies of TLR1pos genotypes than the old group from Germany. The data show that tumor-necrosis-factor (TNF)α, CXCL8 and CCL2 levels were higher in old donors from Germany than in plasma levels from old Italian donors. TNFα and CCL2 levels were significantly raised in old German individuals compared to Italian nonagenarians. German and Italian donors with the TLR1neg genotype basically produced more CCL2 than older European donors with TLR1pos genotypes.

**Conclusion:**

The higher frequency of the TLR1pos genotype in elderly Italian subjects may result from different ethnic populations. Lower inflammatory mediator release of aged Italian individuals is probably due to different background in nutrition, diet, genetics and to psychological aspects. Elderly donors carrying TLR1pos genotypes basically release more anti-inflammatory IL-1Ra and less inflammatory CCL2 suggesting a decline of the pro-inflammatory status found in ageing and, therefore, this may define an anti-inflammatory phenotype. Future studies are needed to elucidate the association of a TLRpos genotype with decreased susceptibility to infections and reduced risk to develop artherosclerosis.

## Background

In human ageing, enhanced morbidity and mortality from infections and declined vaccine responses is reported [1]. This is mainly a consequence of immunosenescence and the functional impairment of the immune system with age [1]. Immunosenescence ascribes the acquired dysregulated immunity within ageing affecting both innate and adaptive immunity. Additionally, increased basal levels of inflammatory cytokines are observed in old individuals, a condition which has been termed “inflammageing” [2]. Changes in numbers and functions of the multiple cell types contribute to the defective innate immunity associated with ageing. However, less is known about the influence of human ageing on TLRs, important components of the innate immune system and sentinels in the recognition of and response to pathogenic microorganisms.

Human TLRs are specific for highly conserved pathogen-associated molecular patterns (PAMPS) of bacteria, fungi and viruses [3,4]. TLR signalling activates a cascade of molecules resulting in the production the pro-and anti-inflammatory cytokines/chemokines and the up-regulation of co-stimulatory molecules. TLRs are differently expressed on a variety of cells, and build a bridge between innate and adaptive immunity by activating dendritic cells and other antigen-presenting cells (APC) [5]. The known ligands for TLRs are nucleic acids (TLR3, 7–9), recognized by intracellular located TLRs [6], and triacylated lipopeptides (TLR1/2), zymosan (TLR2/6), lipopolysaccharide (LPS) (TLR4) and flagellin (TLR5) bound by TLRs located on the cell membrane [7,8].

TLR1/TLR2 heterodimers play an important role in host defense against mycobacterial infections choosing them as attractive targets since tuberculosis is increasing in developed countries with age [9-11]. Within the TLR1 gene, two relevant polymorphisms, the nonsynonymous SNPs T1805G (I602S, rs5743618) and A743G (S248N, rs4833095), are described to be related to mycobacterial derived diseases [8,12]. Both SNPs are suggested to be also associated with leprosy and sepsis [13,14]. Several reports demonstrated that TLR1 genotypes 743AA/1805GG (TLR1neg) are associated with impaired TLR1 cell surface expression and function while heterozygous 743AG/1805TG and homozygous 743GG/1805TT genotypes (TLR1pos) positively correlate with TLR1 cell surface expression [8,13,15,16].

First studies of TLRs in the context of ageing were performed in mice. TLR1-9 expression and function have been reported to be generally impaired in macrophages from aged mice [17]; a functional difference has also been described. In contrast, others could not confirm changes in murine TLR2 or TLR4 surface expression but detected a decrease in pro-inflammatory cytokine production after stimulation [18]. In human studies, no differences in TLR2 and TLR4 surface expression and LPS stimulated cytokine production of monocytes between young and old groups could be observed [19-23]. One group reported that defects in TLR1/2 may result in a higher risk of infection and weak vaccine response [24,25]. The authors detected that, in contrast to young subjects, monocytes from old individuals displayed a lower TLR1 cell surface expression accompanied by a reduced production of TNFα and IL-6 and decreased up-regulation of CD80 after specific TLR1/2 stimulation [25].

Since TLR1 polymorphisms are associated with mycobacterial infections and TLR1 surface expression, the present study was addressed to investigate the influence of these polymorphisms on human ageing. The frequency of TLR1 SNPs 743 and 1805 of healthy old German individuals were compared with young ones and also investigated in old donors and nonagenarians from Italy. Furthermore, the impact of TLR1neg and TLR1pos genotypes on basal cytokine and chemokine production of elderly subjects was examined.

## Results

### Distribution of TLR1 SNP A743G and SNP T1805G frequencies in young and old individuals from Germany

In a previous study [8], it has been shown that the TLR1neg genotype 743AA/1805GG was associated with higher susceptibility to develop tuberculosis. To determine whether TLR1 genotypes are correlated with longevity and ageing, young and old individuals from Germany were analyzed. A tendency to a decreased homozygous TLR1pos 743GG/1805TT genotype and a higher accumulation of the TLR1neg genotype 743AA/1805GG in the old German individuals could be detected (Table 1); though, these results were statistically not significant (p = 0.097; p = 0.081, Fisher’s exact test). In summary, these data reveal no evidence for a role of genetic TLR1 variants in ageing and lifespan referred to the German population.

**Table 1:** Comparison of the frequency of TLR1 genotypes in young and old individuals from Germany

**A.** TLR1 A743G genotype	Young (n = 60)	Old donors (n = 101)	p-value (Fisher’s exact test)
AA	33	64	
AG	22	35	0.604
GG	5	2	0.097
AG + GG	27	37	0.321
**B.** TLR1 T1805G genotype	Young (n = 60)	Old donors (n = 101)	p-value (Fisher’s exact test)
GG	30	55	
TG	24	43	0.135
TT	6	3	0.081
TG + TT	30	46	0.626

### Comparison of the distribution of TLR1 SNP A743G and SNP T1805G frequencies in elderly individuals from Germany and Italy

In order to analyze and compare the frequencies of TLR1 genotypes in different European populations (Italian aged group and old German group), a significant shift to the TLR1pos genotype was noticed in the Italian group (Table 2). The old donors from Italy predominantly carried the heterozygous TLR1 743AG/1805TG genotype and displayed only 26% (743AA) and 19% (1805GG) TLR1neg genotypes. This was in contrast to old German subjects carrying the TLR1neg genotype more than over 50%. The old group from Italy also revealed significantly higher frequencies of the homozygous TLR1pos 743GG and 1805TT (p = 0.000001) genotypes compared to the old group from Germany (Table 2). The same was detected when comparing old individuals from Germany and nonagenarians from Italy (data not shown).

**Table 2:** Comparison of the frequency of TLR1 genotypes in old individuals from Germany (G) and Italy (I)

**A.** TLR1 A743G genotype	Old (G) (n = 101)	Old (I) (n = 50)	p-value (Fisher’s exact test)
AA	64	13	
AG	35	24	0.003
GG	2	13	0.000001
AG + GG	37	37	0.00003
**B.** TLR1 T1805G genotype	Old (G) (n = 101)	Old (I) (n = 53)	p-value (Fisher’s exact test)
GG	55	10	
TG	43	24	0.009
TT	3	19	0.000001
TG + TT	46	43	0.00002

To investigate whether the different distribution among old individuals in Germany and in Italy resulted from age or from ethnical differences, the TLR1 SNP A743G genotype frequencies of the old persons from Italy (n = 50–53; age 66–89) to nonagenarians from Italy (n = 31–32; age 90–99) were compared (Table 3). No significant difference between these two groups (743AG: p = 0.157; 743GG: p = 0.486; Fisher’s exact test) was found. The same result was derived comparing the same groups in SNP T1805G (TG: p = 0.164; TT: p = 0.179) (Table 3). This suggests that the difference between old individuals from Germany and Italy is a result of different ethnic population characteristics and not of the higher mean age in the Italian group.

**Table 3:** Comparison of the frequency of TLR1 genotypes in old and nonagenarian individuals from Italy

**A.** TLR1 A743G genotype	Old (n = 50)	Nonagenarians (n = 31)	p-value (Fisher’s exact test)
AA	13	4	
AG	24	19	0.157
GG	13	8	0.486
AG + GG	37	27	0.260
**B.** TLR1 T1805G genotype	Old (n = 53)	Nonagenarians (n = 32)	p-value (Fisher’s exact test)
GG	10	2	
TG	24	16	0.164
TT	19	14	0.179
TG + TT	43	30	0.197

When the frequencies of these TLR1 genotypes in the old and a nonagenarian group from Italy were compared, no significant distributions could be observed. Interestingly, a very similar contribution of SNP A743G and T1805G was detected in the Italian groups. This observation has also been described in studies in which comparing different ethnic populations had been compared [8,15], indicating that both TLR1 SNPs are in linkage disequilibrium [8,15].

### Basal cytokine expression of peripheral blood mononuclear cells (PBMC) isolated from old donors with TLR1neg 743AA and TLR1pos 743AG genotypes

**Figure 1:**
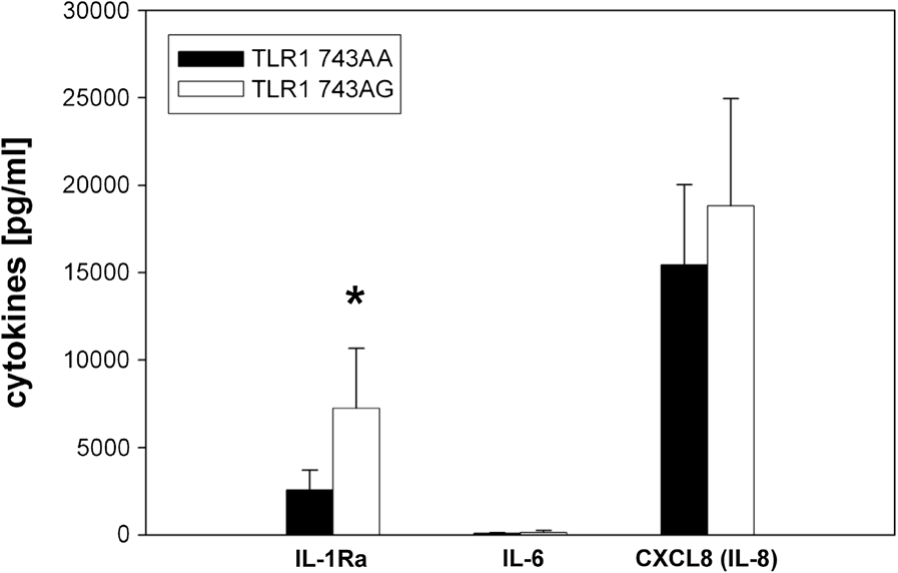
**Basal IL-1Ra, IL-6 and CXCL8 production of PBMC from healthy elderly subjects with different TLR1 genotypes.** PBMC isolated from healthy elderly donors with the TLR1neg genotype 743AA and TLR1pos genotype 743AG were incubated for 72 hours in culture medium, followed by the collection of cell-free supernatants. IL-1Ra, IL-6 and CXCL8 concentrations were determined by ELISA. Student’s t-test, two-tailed, p = 0.0031; TLR1 743AA, n = 4, TLR1 743AG, n = 6.

IL-1Ra is an anti-inflammatory cytokine playing a role in infectious diseases, in dissolving inflammatory reactions [26], and in ageing [27]. The basal expression of IL-1Ra, IL-6 and CXCL8 (IL-8) of PBMC isolated from 4 individuals with the TLR1neg genotype 743AA and 6 with the genotype 743AG [8] was measured by ELISA (Figure 1). These old individuals were participants of the ZINCAGE study [28]. None of the donors carried the TLR1pos 743GG genotype.

No significant difference in the production of IL-6 and CXCL8 between the AA and AG genotypes of the SNP 743 could be detected, but IL-1Ra was significantly increased in old individuals with the TLR1pos genotype AG (Figure 1; p = 0.031, Student’s t-test).

### Comparison of basal cytokine and chemokine production of aged individuals from Germany and Italy

**Figure 2:**
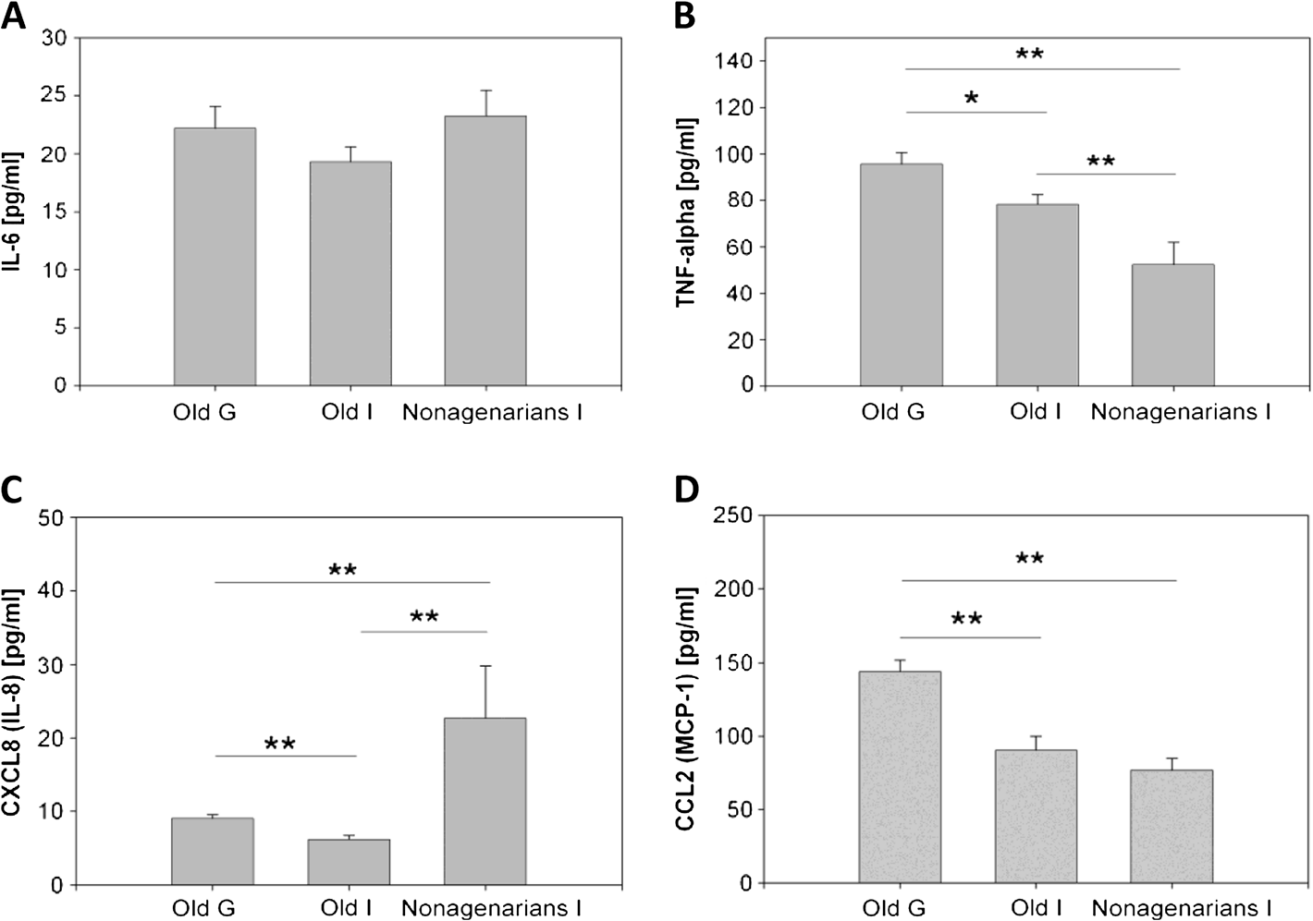
**Plasma levels of cytokines/chemokines derived from old individuals from Germany and Italy and from Italian nonagenarians.** Analyses were performed as described in Methods. Old (G), n = 75-90; old (I), n = 38-40; nonagenarians (I), n = 14-24. G, Germany; I, Italy. Wilcoxon statistic test, mean + standard error, *p <0.05; **p < 0.01.

Aged individuals show increased serum levels of inflammatory cytokines (IL-1β, IL-6, TNFα, IL-1Ra) and chemokines such as CXCL8 and CCL2 (MCP-1) [29-35]. The analyses of the plasma levels of old individuals from Germany and Italy as well as from nonagenarians from Italy revealed that there were no differences in basal IL-6 levels between the different ageing groups (Figure 2A). Constantly, the old donors from Germany had significantly higher levels of basal TNFα, CXCL8, and CCL2 than the old people from Italy, and higher concentrations of TNFα and CCL2 than the nonagenarians from Italy (Figure 2B-D). Interestingly, the samples from the nonagenarian group significantly contained higher CXCL8 but lower TNFα concentrations than the old groups from Germany and Italy (Figure 2B, C).

### Comparison of basal plasma cytokine and chemokine levels of aged donors with TLR1neg 743AA/1805GG and TLR1pos 743AG/1805 T, 743GG/1805TT genotypes

**Figure 3:**
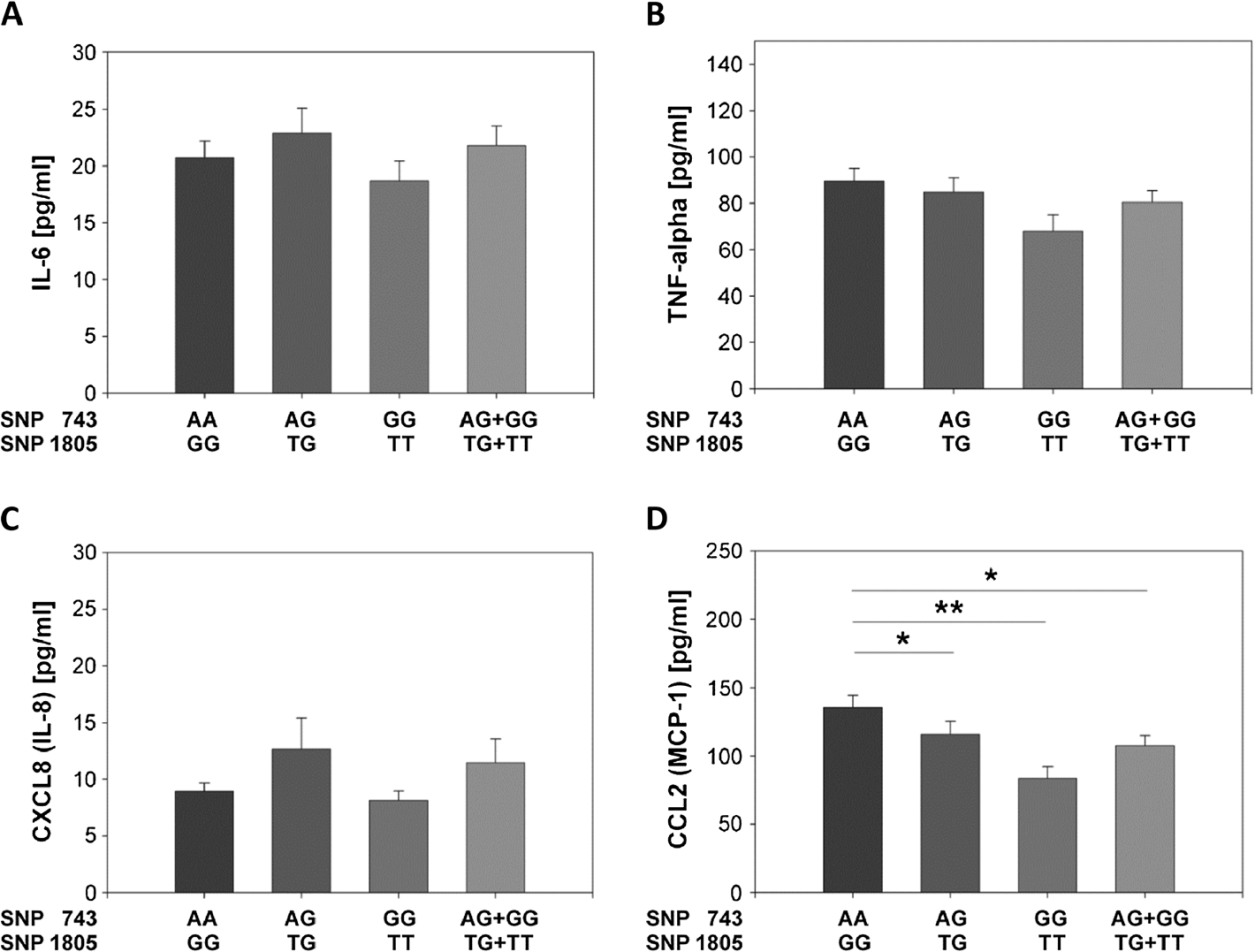
**Comparison of cytokine/chemokine plasma concentrations of aged donors with different TLR1 genotypes.** Analyses were performed as described in Methods. The plasma cytokine concentrations were compared between a homozygous TLR1neg genotype group (743AA/1805GG), a heterozygous TLR1pos genotype group (743AG/1805GT), a homozygous TLR1pos genotype group (743GG/1805TT), and all TLR1 positives (743AG/1805GT and 743GG/1805TT), statistically compiled from old individuals from Germany and Italy and from nonagenarians (Italy). 743AA/1805GG genotype, n = 66–76; 743AG/1805GT genotype, n = 65-66; 743GG/1805TT genotype, n = 23; 743AG/1805GT and 743GG/1805TT genotypes, n = 88-89. Wilcoxon statistic test, mean + standard error, *p <0.05; **p < 0.01.

Since higher levels of pro-inflammatory cytokines are found in aged subjects and TLR1 deficiency is accompanied with delayed cytokine production [25,36], it was examined whether different TLR1 genotypes are associated with different plasma cytokine/chemokine levels in ageing. All old individuals from Germany and Italy and nonagenarian from Italy were sorted into a TLR1 negative genotype (743AA/1805GG) group, a heterozygous TLR1pos 743AG/1805TG group and a homozygous TLR1pos 743GG/1805TT group. Next, the basal cytokine and chemokine levels between the different TLR1 genotype groups were analyzed and compared. No differences in the basal expression of IL-6, TNFα and CXCL8 between the different TLR1 genotypes could be detected (Figure 3A-C). In contrast, circulating levels of CCL2 were significantly lower in the TLR1pos 743AG/1805TG as well as in the 743GG/1805TT group compared to the TLR1neg 743AA/1805GG group (Figure 3D). These data indicate that elderly individuals with a TLR1pos genotype produce less inflammatory CCL2 which may decrease the activation and the recruitment of monocytes.

## Discussion

The association of TLR polymorphisms with diseases and greater susceptibility to infections has been reported [37]. As known from many studies, TLR1 is an essential component in the defence against *Mycobacterium tuberculosis, Mycobacterium leprae, Borrelia burgdorferi* and *Neisseria menigitidis*[14,38]. Consequently, a defect in TLR1 expression and function may result in an impaired immune response against these and other microorganisms in aged individuals [39]. Since associations of the TLR1 SNP A743G and T1805G with higher susceptibility to leprosy, tuberculosis and sepsis have been demonstrated [8,12,13,40], TLR1 polymorphisms may also have clinical implications for aged subjects.

In ageing, defects in TLR1/2 expression and signaling are potentially associated with higher risk of infection and poor vaccine responses, e.g. to influenza [24,39]. Van Duin et al. [25] reported that after specific TLR1/2 stimulation, monocytes from older individuals produced half as much TNFα and IL-6 and displayed a reduced up-regulation of CD80 than younger ones. In contrast, no differences between young and old monocytes were found after specific activation of TLR2/6, TLR4 or TLR5. This defect in TLR1 was accompanied by a 36% lower TLR1 surface expression in the old subjects compared to young individuals, whereas TLR2 expression was unchanged [25]. But this study was restricted to the analysis of one cell type only, and was not performed under the criteria of SENIEUR, a protocol for studying healthy aged persons [41]. Surprisingly, questions or experiences addressing the possibility that low or no TLR1 surface expression have been resulting from genetic TLR1 variants were missing, also in follow-up studies [24]. Based on our data [8] and the data presented herein, it can be hypothesized that the TLR1 SNPs A743G and T1805G partially account for TLR1 defects observed in older subjects.

However, the role of TLR polymorphisms in ageing is incompletely understood. One study examined the TLR4 SNP A896G (D299C), associated with decreased LPS stimulated cytokine synthesis, in subjects with acute myocardial infarction (AMI), young people and centenarians [42]. The data showed significant differences in the SNP frequency between patients with AMI and centenarians. Additionally, the frequency of this polymorphism was significantly higher in centenarians compared to healthy young controls. This may indicate that SNPs related to impaired TLR responsiveness are associated with longevity, but, on contrary, SNPs can also be associated with higher susceptibility to infections [reviewed in [39].

Our data revealed that genetic variants of TLR1 were not associated with longevity, but there was a tendency that old people from Germany more frequently express the TLR1 negative genotype 743AA/1805GG and less the homozygous TLR1pos genotype. Further studies with larger numbers of old and young individuals will finally define whether a specific TLR1 genotype contributes to larger lifespan or susceptibility to tuberculosis or not.

It could be shown that PBMC from old German individuals with the TLR1pos 743AG genotype released significantly more anti-inflammatory IL-1Ra than aged individuals with the TLR1neg 743AA genotype (Figure 1). There are different reports about the circulating IL-1Ra concentrations in the elderly. The serum/plasma levels of IL-1Ra tend to be elevated with normal ageing [33,43], others found no differences between young and old individuals [44]. Ferrucci and coworkers did not observe a correlation between age and IL-1Ra after adjusting for cardiovascular risk factors, additionally [29]. On contrary, it has been reported that high levels of plasma IL-1Ra, along with IL-6 and C-reactive protein (CRP), were predictive of higher mortality in nonagenarians [27]. Our results show evidence that elevated IL-1Ra levels of old TLR1pos subjects could dampen the basal pro-inflammatory status of the elderly by inhibiting the binding of IL-1 to the IL-1 receptor I. Therefore, this suggests that aged TLR1pos genotype carriers are associated with an anti-inflammatory phenotype.

In contrast to young and old German individuals, old people and nonagenarians from Italy basically carry the TLR1 positive genotypes, with highest frequency of the heterozygous 743AG/1805TG genotype. This might be due to variable TLR1 SNP A743G and T1805G frequencies in different ethnic populations. European-Americans, African-Americans, Sub-Saharan-Africans mainly have the homozygous TLR1pos genotype [8,15]. In different European populations only a very few frequency of the homozygous TLR1pos genotype can be observed and more than 50% of the Europeans carry the TLR1neg743AA/1805GG genotype (National Center of Biotechnology Information (NCBI), database SNP; Reference SNP cluster reports for rs4833095 and rs5743618; http://www.ncbi.nlm.nih.gov/snp/). The cause why variable TLR frequencies exist in different ethnic populations is not known. Along with founder effects and migration patterns of ancestors, selective pressures from diseases are discussed [18].

Ageing is accompanied by a chronic low-grade inflammation characterized by 2-4-fold increase in serum levels of inflammatory mediators [31,45], potentially triggering the onset of age-related inflammatory diseases. TNFα, CXCL8, and CCL2 plasma levels were found to be lower in old Italians and nonagenarians than in the plasma of old Germans (Figure 2). Explanations for this discrepancy could be: different genetic backgrounds, zinc status, psychological dimensions (cognitive functions, mood, perceived stress), body mass index (BMI) and nutritional aspects [46]. Additionally, different diets between German and Italian donors [47], and different plasma cytokine levels between ethnic populations [44] may contribute to the observed varieties in cytokine/chemokine levels.

Plasma levels of TNFα raise with increasing age and are associated with morbidity and mortality in elderly populations [34,35]. Nonagenarians from Italy displayed lower TNFα levels than the other old groups (Figure 2). That may fit into the results of a study in which increased T lymphocytes amounts from 81 year old subjects contribute to elevated TNFα, levels, while centenarians did not show this altered TNFα secretion profile [48]. Interestingly, nonagenarians had significantly higher levels of CXCL8 than old people from Germany and Italy. CXCL8 seems not to be elevated in the serum of the elderly [49]. Other studies reported increased levels of CXCL8 and enhanced basal production by monocytes, which may be responsible for chronic inflammatory rheumatic diseases in combination with pro-inflammatory cytokines, [32,50]. These observations have to be confirmed by other studies to elucidate whether enhanced CXCL8 leads to larger recruitment of neutrophils to effectively combat an infection or to boost the existing low-grade inflammation, additionally.

Aged donors from Italy and Germany with TLR1pos genotypes had significantly less CCL2 plasma levels than donors with the TLR1neg genotype (Figure 3D). CCL2 increases during human ageing probably due to dynamic changes of circulating monocytes [30,51]. Among other chemokines and chemokine receptors, CCL2 contributes to the development of artherosclerosis [52], is upregulated in animal models of mycocardial ischemia and highly expressed in artherosclerotic lesions. In addition, aged aortic vascular smooth muscle cells (VSMC) basically express more IL-6, TLR4, and CCL2 than young VSMC in a murine *ex vivo* culture system [52,53]. Others described that increasing CCL2 levels with age are independently associated with artherosclerosis [54].

The data indicate that elderly with a TLR1pos genotype produce lower inflammatory CCL2 which may lead to a reduced recruitment of monocytes and decreased inflammatory conditions. Combined with elevated IL-1Ra, aged individuals with a TLR1pos genotype may have a lower risk to develop artherosclerosis, but further studies are needed to support this hypothesis.

In particular, modification of zinc plasma concentration has been found in aging and in some age-related diseases [46]. Since ageing and zinc deficiency are characterized by impaired immune responses, oxidative stress and low grade chronic inflammation, a relationship between both states has been hypothesized [46,55]. Additionally, cytokine genes are highly polymorphic and for example the IL-6 SNP -174 G/C polymorphism appears to be associated with age-related diseases characterized by an impaired zinc status [56]. In context with our data, carriers with TLR4 SNP +896 G + genotype expressed lower levels of IL-6 and TNFα and higher level of anti-inflammatory IL-10 [45]. These cytokines are involved in artherosclerosis and longevity. Thus, TLR4 SNP +896 G + seems to determine a minor risk to develop atherosclerosis by decreasing pro-inflammatory signals and increasing anti-inflammatory cytokine production [45,57]. Therefore, the TLR1pos genotype associated with a positive control of inflammation may also play a protective role against atherosclerosis and promote longevity. Inasmuch zinc status and TLR1 polymorphisms are related to longer lifespan and control of age-associated diseases have to be shown in future approaches.

## Conclusions

In our study, only a tendency that old German donors more frequently carry the TLR1neg genotype and less the TLR1pos homozygous genotype than young subjects was observed. In contrast, old and nonagenarian individuals from Italy show significantly higher frequencies for the TLR1pos genotype indicating a possible lower risk for mycobacterial infections.

The observation that old and nonagenarian individuals from Italy produce lower basal levels of TNFα, CXCL8 and CCL2 than aged German donors may be due to distinct genetics, zinc status, different nutritional and psychological backgrounds.

TLR1pos aged donors show an anti-inflammatory phenotype characterized by a higher IL-1Ra production and a reduced release of CCL2. In combination, these factors antagonize the pro-inflammatory milieu present in ageing.

In summary, aged individuals with a TLR1pos genotype may have a reduced risk to develop artherosclerosis, tuberculosis and other chronic inflammatory diseases than aged TLR1neg individuals. Hence, this needs to be proven in future studies.

## Methods

### Young and elderly donors

A total of 101 (72 men, 29 women) physically and mentally healthy volunteers aged 65–82 (mean age of 70.0 years), were recruited in Aachen, Germany, and designated as the old group. Aged subjects were drawn from middle class independently living healthy volunteers between the ages of 65 years and 88 years. A detailed questionnaire was used to exclude any significant illness, i.e. metabolic syndrome (diabetes/cardiovascular disorders), infections, malignancy. A second old group (n =53; 26 women, 27 men), consisted of individuals aged 66–77 (mean age 70.5 years) recruited from Italy; the third group n = 33 (13 men, 20 women) were aged 90–99 (mean age 92.8; nonagenarian group) from Italy as well. The total mean age of all Italian individuals was 79.3 years. All individuals fulfilled the criteria of the SENIEUR protocol [8,28,41] and qualification and selection criteria required by the European specific targeted research project ZINCAGE (http://www.zincage.org) [28].

The group of young donors consisted of 60 healthy individuals, (20 men, 40 women), 57 were 22–35 years old, three individuals out of 60 were 40, 40 and 46 years old (mean age 29); all were physically and mentally healthy and did not take any acute or chronically medication for any disease.

The DNA samples were gained from whole EDTA blood, fresh or frozen at −20°C, using the Qiagen kit (Hilden, Germany) according to the manufacturer’s instructions. Isolated PBMC (peripheral blood mononuclear cells) from ten physically and mentally healthy elderly volunteers aged 65–82 years from Aachen, Germany, described before [28], were used for basal expression studies of cytokines.

The circulating concentration of selected chemokines CCL2, CCL3, CXCL8, and cytokines IL-6 and TNFα were measured in plasma obtained from groups of healthy old subjects from Germany and Italy; which were participants of the ZINCAGE study [32]. The study was approved by the Ethics committee of the Medical Faculty, RWTH Aachen University, Germany (EK 061/04 and EK 023/05).

### Single nucleotide polymorphism (SNP-) Real-time PCR assay

DNA was extracted from whole blood using the DNAamp kit (Qiagen, Hilden, Germany). Primers and probes for the discrimination of TLR1 positives (homozygous GG, heterozygous AG), and negatives (heterozygous AA) at position 743 were used and the SNP-PCR assay was performed as described recently [8] on a 7000 SDS (Applied Biosystems, Foster City, CA, USA). For SNP detection of TLR1 T1805G (I602S), a restriction fragment length polymorphism (RFLP) PCR was performed using the primer pairs described by Johnson et al. [13] followed by *Pst*I digestion of the PCR products.

### Cytokine expression

CCL2, CXCL8, IL-6 and TNFα were analyzed in plasma samples of the aged from Germany and Italy and assayed in multiplex beads assays as described by Mariani et al. [32]. Culture conditions and stimulation of PBMC from the ten donors were performed as described previously [28]. For quantification of hIL-1Ra (R&D systems, Wiesbaden, Germany), hIL-6, hIL-8, antibody sets (all BD Biosciences, San Jose, CA, USA) were used. ELISAs were quantified using an ELISA-plate reader (Sunrise by Tecan Austria, Salzburg, Austria).

### Statistical analysis

Continuous variables are presented as mean and standard deviation (SD). Categorical data are presented by frequencies and percentages. Associations of alleles with disease or genotype with disease were represented in 2 × 2 contingency tables and analyzed using Fisher’s exact test.

All tests were two-tailed and assessed at the 5% significance level. Because of the exploratory nature of the analyses, no adjustment was made to the significance level to account for multiple testing. Analyses were performed as described [8].

Statistical significance of experimental results (cytokines, chemokines) was calculated by Student’s *t*-test and Wilcoxon signed-rank test using GraphPad Prism version 5.00 for Windows, GraphPad Software (San Diego, CA, USA).

## Competing interests

The authors declare they have no competing interests.

## Authors’ contribution

PU designed the experiments, analyzed data, did statistical analysis, and wrote the manuscript; EMO performed experiments and analyzed data; EM, MM, and EM collected samples, designed and performed experiments and analyzed data; and LR designed the project, analyzed data, and supervised the work. All authors read and approved the final manuscript.
